# Structural and process controls of AIEgens for NIR-II theranostics

**DOI:** 10.1039/d0sc02911d

**Published:** 2020-06-12

**Authors:** Shunjie Liu, Yuanyuan Li, Ryan T. K. Kwok, Jacky W. Y. Lam, Ben Zhong Tang

**Affiliations:** Department of Chemistry, The Hong Kong Branch of Chinese National Engineering Research Center for Tissue Restoration and Reconstruction, Institute for Advanced Study, Department of Chemical and Biological Engineering, Division of Life Science, State Key Laboratory of Molecular Neuroscience, The Hong Kong University of Science and Technology Clear Water Bay Kowloon Hong Kong China tangbenz@ust.hk; Center for Aggregation-Induced Emission, SCUT-HKUST Joint Research Institute, State Key Laboratory of Luminescent Materials and Devices, South China University of Technology Guangzhou 510640 China

## Abstract

Aggregation-induced emission (AIE) is a cutting-edge fluorescence technology, giving highly-efficient solid-state photoluminescence. Particularly, AIE luminogens (AIEgens) with emission in the range of second near-infrared window (NIR-II, 1000–1700 nm) have displayed salient advantages for biomedical imaging and therapy. However, the molecular design strategy and underlying mechanism for regulating the balance between fluorescence (radiative pathway) and photothermal effect (non-radiative pathway) in these narrow bandgap materials remain obscure. In this review, we outline the latest achievements in the molecular guidelines and photophysical process control for developing highly efficient NIR-II emitters or photothermal agents with aggregation-induced emission (AIE) attributes. We provide insights to optimize fluorescence efficiency by regulating multi-hierarchical structures from single molecules (flexibilization) to molecular aggregates (rigidification). We also discuss the crucial role of intramolecular motions in molecular aggregates for balancing the functions of fluorescence imaging and photothermal therapy. The superiority of the NIR-II region is demonstrated by fluorescence/photoacoustic imaging of blood vessels and the brain as well as photothermal ablation of the tumor. Finally, a summary of the challenges and perspectives of NIR-II AIEgens for *in vivo* theranostics is given.

## Abstract 2

Structural and process controls of NIR-II AIEgens realize manipulating of radiative (R) and nonradiative (NR) decay for precise theranostics.

## Graphical abstract

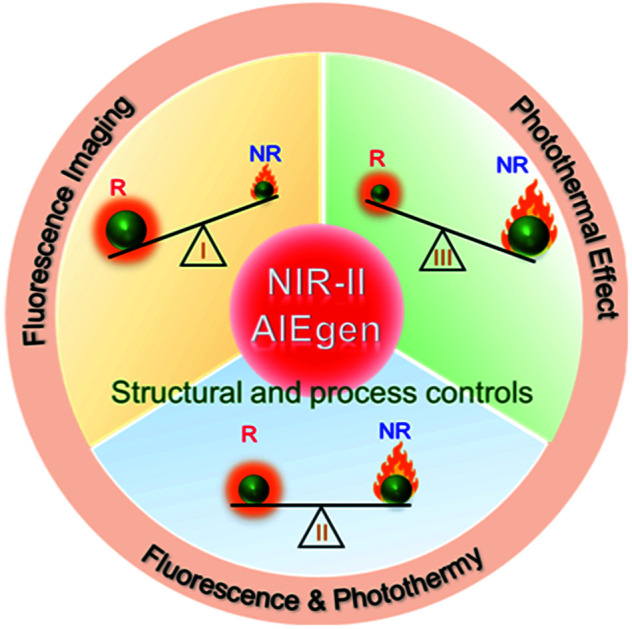

## Introduction

**Fig. 1:**
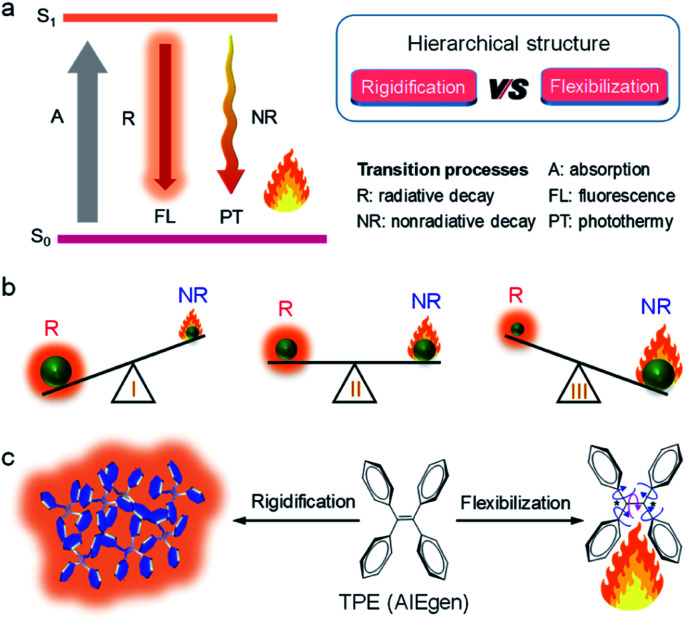
(a) Jablonski diagram. (b) Schematic illustration of the competition between radiative decay (R) and nonradiative decay (NR). (c) Graphic description of the AIE mechanism.

Light is one of the most fundamental branches of science. It is essential to decipher the interaction between light and matter for promoting the advances in technologies associated with the utilization of light, such as fiber-optic communication, optical storage, optical devices, *etc.* Recent years have witnessed the establishment of strong ties between light and life by being able to visualize and detect many previously unknown life processes. Understanding photophysical behaviors is crucial for the design and interpretation of light-based applications. According to the classical photophysical diagram (Fig. 1a), matter absorbs light energy into excited energy states before immediately falling back to lower energy states mainly through radiative (R) and non-radiative (NR) decays.^1^ R is mostly in the form of fluorescence, which can be utilized for bioimaging and biosensing. Heat generation is the main outcome of NR, which has numerous applications in photothermal therapy (PTT), photoacoustic (PA) imaging, laser resurfacing, desalination of seawater, *etc.*^2,3^ Unfortunately, these two critical processes compete against one another: the dominant R will suppress NR and *vice versa*. So how can we regulate these two processes to attain desirable properties?

Among all factors affecting the process of R and NR, the structure is undoubtedly one of the most significant aspects (Fig. 1a, right). Generally, matter with rigid structures shows a dominated R, resulting in a strong fluorescence (Fig. 1b, left). In contrast, flexible matter favors molecular motion for an overwhelming NR, giving an intense heat generation (Fig. 1b, right). Accordingly, simultaneous introduction of rigidification and flexibilization into structures endow matters with both fluorescence and heat (Fig. 1b, middle). But how should we regulate the structure? Conventionally, structural regulation at the molecular level can precisely control molecular properties. For example, some molecules with rigid fused-ring structures show strong fluorescence as isolated species. However, when they aggregate in life mediums such as water, the resultant higher-order hierarchical structure exhibits fluorescence quenching, owing to the excimer formation. This is the so-called aggregation-caused quenching (ACQ) phenomenon, which severely hinders *in vivo* bioimaging.^4,5^ On the other hand, some flexible molecules, such as tetraphenylethylene (TPE), are almost non-emissive at a molecular level, owing to the dominated NR by active intramolecular motions (Fig. 1c).^6–8^ Upon molecular aggregation in water, intermolecular hydrophobic interactions restrict the intramolecular motions, giving a significantly enhanced fluorescence. Therefore, the rigidification of molecular structures at a higher-order hierarchical level gives a much stronger fluorescence intensity than that of the molecular level. Altogether, by regulation of hierarchical structures (flexibilization at a molecular level and rigidification at a morphological level), the same substance may display predesigned photophysical properties to meet various applications.

**Fig. 2:**
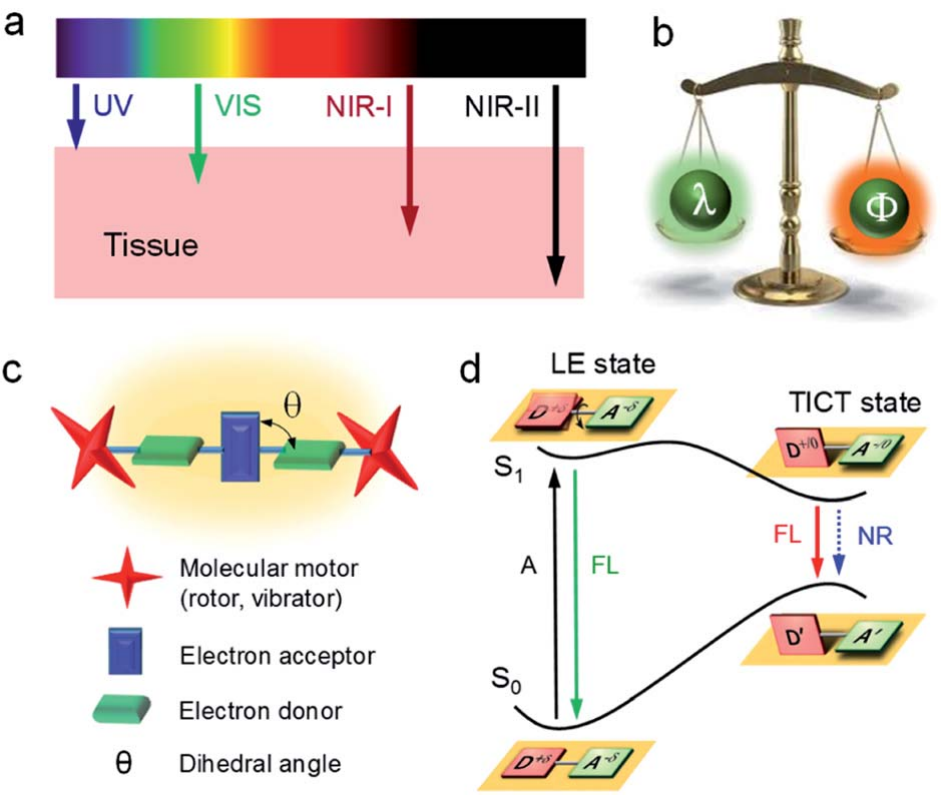
(a) Schematic tissue penetration depth of light with different wavelengths. (b) The balance between emission wavelength (*λ*) and efficiency (*Φ*). (c) The molecular design strategy of NIR-II AIEgens. (d) Jablonski diagrams of TICT dynamics.

Fluorescence imaging exhibits enormous potential to visualize and detect disease-related processes and has been successfully used in many clinical cases.^9–13^ Notably, compared with visible (400–680 nm) and first near-infrared region (NIR-I, 700–900 nm), fluorescence imaging in the second near-infrared wavelength region (NIR-II, 1000–1700 nm) shows salient advantages of deep tissue penetration with high signal-to-background ratio (SBR), owing to the decreased scattering of photons at longer wavelengths (Fig. 2a).^14–28^ However, the fluorescence efficiency (*Φ*) decreases remarkably with wavelength (*λ*), especially in low energy bandgap NIR-II materials, as intramolecular motions around the single or double bond result in dominant non-radiative relaxation dynamics (Fig. 2b).^29–32^ Thereof, ways to increase *Φ* of long-wavelength NIR-II fluorophores remains challenging.

Aggregation-induced emission (AIE) is an inventive fluorescent technique and has the potential to address this issue.^4,33–51^ AIE illustrates a unique photophysical phenomenon where molecules are non-emissive in the discrete state because of active intramolecular motions, but emit energetically when aggregated due to the restriction of intramolecular motions (RIM). To drive emission to the NIR-II window, AIE luminogens (AIEgens) are designed as conjugated donor–acceptor (D–A) structures, where they are being twisted with each other (dihedral angle of *θ*, Fig. 2c). Under a polar environment, the intramolecular rotations of D/A units bring the molecules from a locally excited (LE) state to a twisted intramolecular charge transfer (TICT) state (Fig. 2d).^52–54^ The elevation of the highest occupied molecular orbital level narrows the energy bandgap, giving a red-shifted emission. On the other hand, owing to the susceptibility of the TICT state to various nonradiative quenching process, the emission intensity is significantly weakened. Thus, the TICT process gives a red-shifted emission and an excellent photothermal property. According to the RIM mechanism of AIE, the partial restriction of intramolecular motion by molecular aggregation can enhance the fluorescence intensity (on a morphological level) while maintaining the TICT property (molecular level). Moreover, introducing molecular motors to molecules further suppresses the intermolecular interactions to trigger the AIE effect. Therefore, NIR-II AIEgens with long-wavelength fluorescence and intense brightness can be seized simultaneously.

Based on the above-mentioned structural design (twisted backbone + molecular motor) and process control (AIE + TICT), many outstanding NIR-II AIEgens have been reported. In this feature review, to understand the structural design, process control, as well as involved mechanisms, we have selected some representative examples to analyze and guide further developments of NIR-II AIEgens. The R and NR channels can be opened or blocked by tuning the structure at multi-hierarchy levels. AIE technology endows molecules with high fluorescence quantum yield (QY), while the TICT state gives molecules with both red-shifted emission and enhanced photothermal property owing to the predominant NR. By controlling structure and process, systems with predominated fluorescence intensity, or predominated photothermal property, or a balance of fluorescence intensity and photothermal property can be obtained. We hope this review will offer valuable guidance on how to design desirable NIR-II theranostic agents by regulating structure and process.

## Design of NIR-II AIEgens

To date, the majority of the existing NIR-II organic chromophores have low quantum yields between 0.3% and 2%.^55–57^ It will be favorable to design fluorophores with inherently longer emission wavelength, as well as much higher QY (>2%) to achieve effective NIR-II fluorescence imaging. Given the diversity of designing D–A conjugated molecules, this may be realized through molecular engineering of electron donors, acceptors, spacers, *etc.*^58,59^ On the other hand, AIE features outstanding solid-state photoluminescence.^44,60^ These AIEgens naturally own twisted architectures dressed up with several molecular motors, which are valuable for suppressing the robust intermolecular interactions. In general, visible light-emissive AIEgens are relatively easy to achieve through the incorporation of twisted molecular motors. However, this method is not fit for NIR-absorbing fluorophores, as they suffer from much stronger intermolecular π–π interactions, which results in fluorescence quenching when dispersed in a physiological environment. To address this issue, the strategy of “backbone distortion + twisted motors” is generally accepted for designing highly bright NIR-II AIEgens because of the efficient restriction of intermolecular interactions.^61^

**Fig. 3:**
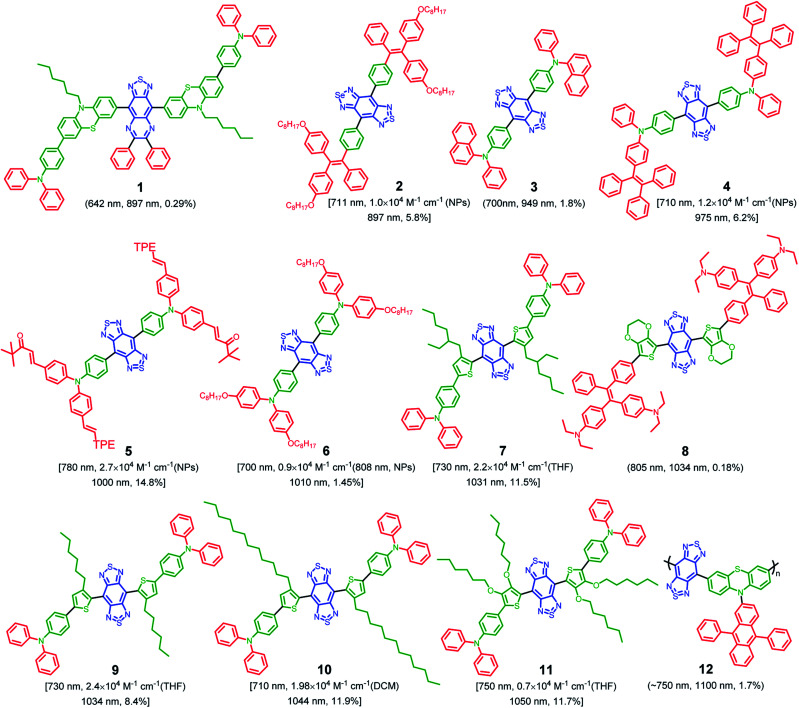
Representative chemical structure of NIR-II AIEgens annotated with absorption maxima, molar absorption coefficient (specific solvent), emission maxima and NIR-II QY in water (1000–1500 nm, based on QY_IR26_ = 0.5%). Note: QY in 1 is calculated based on IR820 as reference (QY = 4.2% in ethanol), absorption in 10 is measured in THF.

Accordingly, NIR-II AIEgens are characterized by a motor–donor–acceptor–donor–motor (M–D–A–D–M) structure. Strong molecular acceptors, such as 6,7-diphenyl-[1,2,5]thiadiazolo[3,4-*g*]quinoxaline (DPTQ) and benzobisthiadiazole (BBTD), are generally utilized to drive the emission wavelength to the NIR-II region.^20^ The energy gap can be decreased by anchoring molecular donors such as phenyl or substituted-thiophene. The critical element in molecular design lies in the distortion of the central D–A–D backbone, which is crucial to restrict the intermolecular interactions. Meanwhile, the peripheral twisted motors such as tetraphenylethylene (TPE) and triphenylamine (TPA) are indispensable. On the one hand, molecular motors are beneficial for further restricting of intermolecular interactions. On the other hand, they provide intramolecular mobility to access the TICT state. To date, based on the above molecular design philosophy, Tang, Qian and Ding,^61–68^ Zheng and Liu,^69^ Lu and Xiao,^70^ Hong,^71^ Chen and Feng,^72^ Liu,^73^ Xiao,^74^ Wu,^75^ Wang and Jiang,^76^ and Wu^77^*et al.* developed several types of highly bright NIR-II AIEgens with emission peak >1000 nm, with representative chemical structures shown in Fig. 3. In this review, we will not go into detail for all the NIR-II AIEgens mentioned but select the most representative ones to illustrate the molecular design philosophy and process control clearly.

## NIR-II AIEgen with high QY (backbone distortion + molecular motor)

**Fig. 4:**
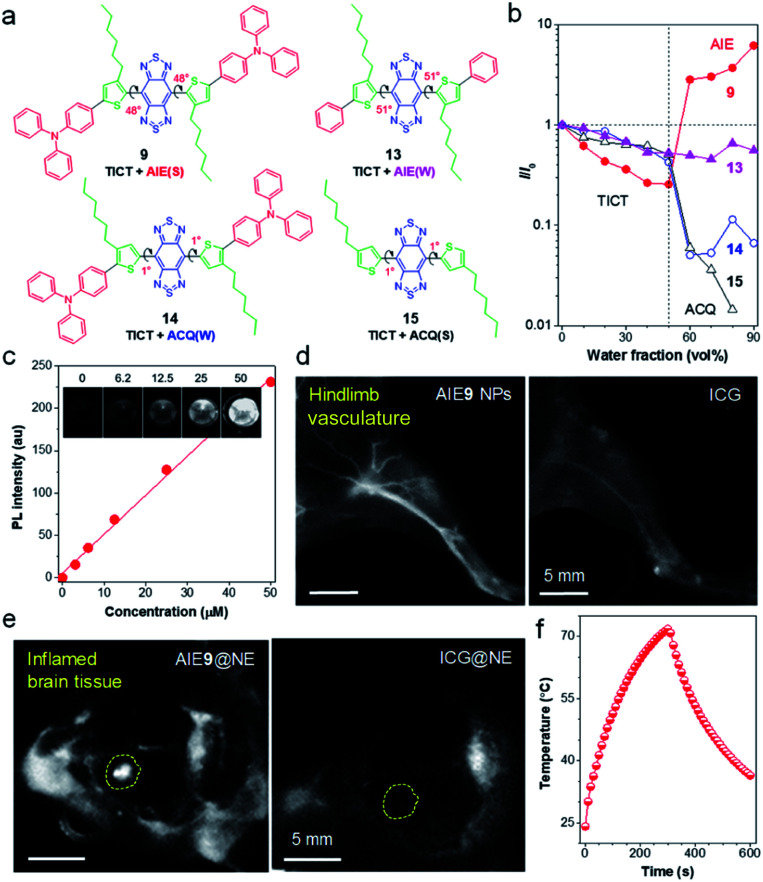
(a) AIEgens with different dihedral angles and molecular motors. Note: “S” and “W” in the bracket represent “strong” and “weak”, respectively. (b) The plot of *I*/*I*_0_ of AIEgens *versus* water fraction in H_2_O/THF mixtures, where *I* and *I*_0_ are the PL intensity of AIEgens in H_2_O/THF mixtures with different water fraction and in THF, respectively. (c) The quantitative relationship between NIR-II fluorescence intensity with concentrations of AIE9 NPs. Inset: corresponding NIR-II fluorescence images. (d) NIR-II fluorescence imaging of blood vessels in hindlimb using AIE9 NPs and ICG, respectively (1000 nm LP, exposure time 50 ms). (e) Noninvasive *in vivo* NIR-II fluorescence images of brain inflammation through intact scalp and skull using neutrophil-carrying AIE9 NPs (AIE9@NE) and ICG (ICG@NE), respectively. (f) The photothermal property of AIE9 NPs under irradiation of 808 nm laser. Data adapted with permission.^61^ Copyright, 2020, Wiley-VCH.

To deeply understand the molecular design strategy, Tang and Ding systematically studied the effects of backbone distortion and molecular motor on the solid-state fluorescence efficiency of the dyes.^61^ As shown in Fig. 4a, because of the existence of steric hindrance in *ortho*-substituted alkylthiophene and BBTD core, AIEgen 9 displayed a significantly distorted backbone (dihedral angle of 48°). Another critical feature of AIEgen 9 was the presence of twisted molecular motor TPA.

Photoluminescence (PL) spectra were performed to detect emission fluctuation upon molecular aggregation using THF/water mixtures (Fig. 4b). Upon increasing water volume fractions (*f*_w_) from 0% to 50 vol%, the fluorescence intensity of AIEgen 9 decreased progressively, due to the forming of a dark TICT state with an increase in polarity. Further increase of *f*_w_ to 90 vol%, the PL intensity showed a rapid enhancement (AIE effect), due to the suppression of intramolecular rotations decisive to the TICT state through molecular aggregations. Therefore, AIEgen 9 possessed a TICT + strong AIE effect with a high QY of 8.4% when in a nanoparticle state. The *α*_AIE_ (PL intensity ratio of *f*_w_ = 90 vol% to *f*_w_ = 0) is also a clear indicator of solid-fluorescence efficiency, with measurements of up to 6.2, which was higher than the other systems (*α*_AIE_ ≈ 2).^71^ To demonstrate the molecular motor effect, AIEgen 13 with phenyl motor while maintaining the highly twisted backbone was used as a control. A typical TICT + AIE effect was observed, however, *α*_AIE_ sharply decreased to 0.54, suggesting a significant impact of the molecular motor on the fluorescence efficiency. On the other hand, the backbone twisting effect was studied by shifting the alkyl chains from *ortho* to *meta* position. In contrast to molecule 9, the resultant molecule 14 adopted a planar backbone (dihedral angle of 1°), owing to the steric distance of *meta*-positioned alkyl chains to the BBTD core. The PL intensity of molecule 14 decreased with *f*_w_ because of the TICT effect upon an increase of polarity. Notably, the emission was almost wholly quenched in the aggregate state (*f*_w_ = 90 vol%), which resulted from the strong intermolecular interactions in the planar D–A–D core. Moreover, removing the twisted TPA motor in molecule 15 further intensified the ACQ effect. Overall, backbone distortion and motor twisting have an equivalent effect on the solid-state fluorescence efficiency of NIR-II AIEgens.

Benefiting from high QY of AIEgen 9 nanoparticles (AIE9 NPs) in the NIR-II region, *in vitro* fluorescence property was examined. As shown in Fig. 4c, a linear relationship between fluorescence intensity with AIE9 NPs concentration was observed, demonstrating the advantages of AIE: the more it gathers, the brighter it glows. To display the *in vivo* fluorescence imaging quality of AIE9 NPs, blood vessel imaging of mouse hindlimb was carried out *via* intravenous tail injection of AIE9 NPs. Indocyanine green (ICG), a NIR-I dye with clinical approval, is served as a comparison. The imaging quality from AIE9 NPs outperformed that of ICG (Fig. 4d), indicating that highly bright NIR-II AIEgens can provide a higher degree of clarity in imaging.

Inflammation is a sign of many major diseases such as cancer, diabetes, cardiopathy *etc.* Noninvasive detection of inflammation is crucial for the early diagnosis of pathogenic processes. In particular, the diagnosis of brain inflammation is viewed as the Holy Grail for bioimaging, as it is well-protected by the blood–brain-barrier (BBB). Tang and Ding ingeniously utilized immune cells, neutrophils (NEs), as “living” carriers to deliver AIE9 NPs to penetrate the BBB to arrive at the brain inflammation site.^61^ As displayed in Fig. 4e, the NIR-II fluorescence signal at the inflamed area injected with neutrophil-carrying AIE9 NPs (AIE9@NE) was much stronger than that of ICG@NE. The signal-to-background ratio (SBR) at the inflammation site reached up to 30.6 for AIE9@NE treated group, while it was only 5.6 for the mice administrated with ICG@NE. Notably, 30.6 is among the one of the highest SBR reported so far. Despite a relatively high QY, it is worth noting that non-radiative decay is still the dominant relaxation pathway in NIR-II materials, as AIE9 NPs displayed excellent photothermal conversion properties (Fig. 4f). Therefore, NIR-II AIEgens have the potential for fluorescence-guided photothermal therapy.

## NIR-II AIEgen with long-wavelength emission (AIE + TICT process)

**Fig. 5:**
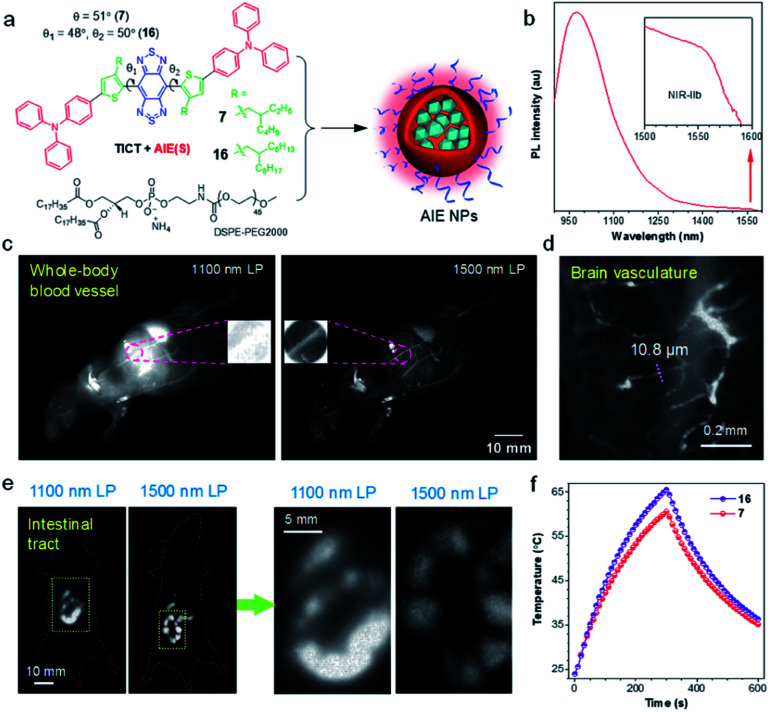
(a) Schematic illustration for the preparation of AIE NPs from AIEgen 7 and 16. (b) PL spectrum of AIE8 NPs. Inset: zoom-in emission spectra in the range of 1500–1600 nm. (c) Comparison of NIR-II fluorescence signal for whole-body imaging of living mice in an area close to liver under 1100 and 1500 nm LP filters, respectively (AIE7 NPs, 200 μL, 0.8 mg mL^−1^). Inset: zoom-in images of the blood vessel. (d) High-magnification through-skull microscopic imaging of the brain vasculature. (e) NIR-II fluorescence images of the intestinal tract using 1100 and 1500 nm LP filters, respectively (AIE7 NPs, 300 μL, 1 mg mL^−1^). Right: zoom-in images of the two yellow rectangle areas. (f) Comparison of the photothermal properties of 7 and 16 NPs. Data adapted with permission.^66^ Copyright, 2020, Nature Publishing Group.

Due to the reduced photon scattering and almost zero autofluorescence, *in vivo* fluorescence imaging in the NIR-IIb region (1500–1700 nm) can provide higher resolution and deeper tissue penetration in comparison with conventional NIR-II window.^78–80^ While inorganic NIR-IIb fluorophores have been developed, the study on organic counterparts is still in its infant, due to the lack of long-wavelength materials. To address this dilemma, Tang and Qian proposed a molecular design guideline for the manipulation of TICT and AIE at molecular and agminated levels, respectively, to explore purely organic NIR-IIb fluorophores.^66^ As shown in Fig. 5a, in line with the molecular design strategy of NIR-II AIEgens, 7 and 16 were synthesized using BBTD as a strong acceptor, with *ortho*-substituted alkylthiophene as the donor, and TPA as the motor. Notably, the keystone of the molecular guidelines is engaged in the selection of secondary carbon-branched alkyl chains, as they supplied a tunable steric barrier not only for restricting intermolecular interactions but also for the activation of intramolecular motions. On a molecular level, the flexible intramolecular rotations of D–A units in AIEgen favored the formation of dark TICT state to yield significantly red-shifted emission wavelength. On the other hand, the NPs formation process (nanoprecipitation with soft amphiphilic polymer DSPE-PEG) partially restricted the intramolecular motions of AIEgens, giving significantly enhanced fluorescence signal. Thereof, dyes with both long-wavelength emission and adequate efficiency can be obtained concurrently by linking ostensibly contradicting individuals. The optimized AIEgen 7 with 2-ethylhexyl unit presented an emission tail extending to 1600 nm with a high NIR-II QY of 11.5% in the nanoparticle state.

To glean the advantage of NIR-IIb fluorescence imaging of AIE7 NPs, long-pass (LP) filters with 1100 and 1500 nm were examined. As shown in Fig. 5c, distinct vascular anatomy with a negligible background is observed in the NIR-IIb window (1500 nm LP), while ambiguous vessels are found in the conventional NIR-II region (1100 nm LP filter). In particular, the vessels can hardly be identified in the conventional NIR-II region for the liver region, owing to the strong autofluorescence of the liver. Through-skull high-magnification microscopic imaging of cerebral vasculature was also carried out. Small vessels with a width of 10 μm could be identified (Fig. 5d). To further demonstrate the merits of penetration depth in the NIR-IIb window, fluorescence imaging of deeply-located intestinal tract was performed. The ileum structure could be distinctly visualized after the gavage of AIE7 NPs at 0.5 h. In contrast to the blurry images observed under 1100 nm LP filter, a clear resolution of tissue features was distinguished in the NIR-IIb region (Fig. 5e). Moreover, NIR-II AIEgen NPs displayed desirable photothermal properties, which were enhanced with elongation of alkyl chains due to the activation of intramolecular motions (Fig. 5f). Therefore, based on structural modulations at molecular (TICT) and agminated levels (AIE), NIR-IIb-based NPs with red-shifted emission wavelength and appropriate QY were attained simultaneously.

Very recently, based on the mechanism of AIE + TICT, Xiao *et al.* developed a series of highly bright NIR-II AIEgen 10 (QY = 11.9%) and 11 (QY = 11.7%) with a fluorescence tail extending to the NIR-IIb region. The resultant AIE NPs could be utilized as excellent NIR-IIb imaging agents for superior spatial–temporal resolution of whole-body, cerebral, tumor vasculature, lymphatic drainage systems, *etc.*^74,81^ On the other hand, NIR-IIa (1300–1400 nm) window is also appealing owing to the reduced light scattering and decreased water absorption. Wu *et al.* reported an AIE-active NIR-IIa conjugated polymer with a high QY of 1.7% in aqueous solution, realizing clear through-skull visualization of the cerebral vasculature.^77^

## NIR-II AIEgen with tunable photothermal therapy (AIE + TICT process)

**Fig. 6:**
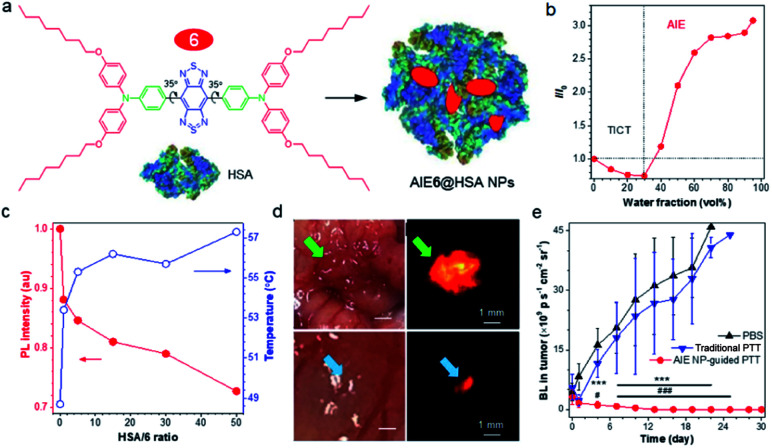
(a) Schematic illustration of AIE6@HSA NPs. (b) The plot of AIEgen 6*versus* water fraction in H_2_O/THF mixtures. (c) The plot of *I*/*I*_0_ (red curve) and temperature (blue curve) of AIEgen 6 in THF : water (5%, v/v) *versus* HSA/6 ratio. (d) Intraoperative NIR-II imaging of orthotopic mouse colon tumor by AIE6@HSA NPs. Upper: a primary tumor in the cecum of the tumor-bearing mouse. Lower: metastatic tumor foci in 0.5 mm × 0.3 mm size. (e) Tumor growth curves of mice bearing orthotopic CT26-Luc colon cancer following different treatment measured by the bioluminescence. Data are presented as mean ± S.D. (*n* = 5). Statistical significance was calculated *via* two-way analysis of variance (ANOVA) with a Tukey *post hoc* test. ***p < 0.001 between the NIR-II image-guided PTT group and PBS group. ^#^*p* < 0.05, ^###^*p* < 0.001 between the NIR-II image-guided PTT group and conventional PTT group. Data adapted with permission.^70^ Copyright, 2019, Nature Publishing Group.

In a D–A-based NIR-II AIEgen, the equilibrium between fluorescence (R) and photothermal effect (NR) is hard to control. To address this issue, Lu and Xiao^70^ reported a strategy of tuning AIE and TICT properties to balance the fluorescence and photothermal effect through the coordination of human serum albumin (HSA) with NIR-II AIEgen 6 (Fig. 6a). In a polar solvent, the active intramolecular rotation drove AIEgen 6 into the dark TICT state, resulting in enhanced NR and photothermal conversion effect (Fig. 6b). On the other hand, when aggregated in poor solvents, restriction of intramolecular motion and suppression of TICT state increased fluorescence (AIE effect). Altogether, the TICT state weakened the fluorescence intensity but elevated the photothermal property. At the same time, AIE increased fluorescence intensity at the cost of its photothermal property, which was balanced by the nature of intramolecular rotations. Enhancement of HSA ratio in the AIE6@HSA NPs activated the intramolecular motions of AIE6 through the coordination with HSA, displaying an enhanced photothermal conversion property at the cost of fluorescence (Fig. 6c). Thus, the prepared AIE6@HSA NPs exhibited both high fluorescence efficiency and high photothermal conversion property, providing high specific imaging of not only primary orthotopic mouse colon tumors but also metastatic lesions with a diameter of 0.5 mm × 0.3 mm (Fig. 6d). Directed by NIR-II fluorescence signal, AIE6@HSA NPs allowed precise PTT of both primary and metastatic lesions, attaining a complete cure of tumors in mice. Considering a large NIR-I (700–900 nm) absorptivity, Zheng and Qian *et al.* also utilized NIR-II AIEgens 4 for *in vivo* photothermal applications, effectively achieving fluorescence-guided photothermal therapy or fluorescence/photoacoustic dual model imaging.^67,69^

## Activation of intramolecular motion for enhanced photothermal therapy

**Fig. 7:**
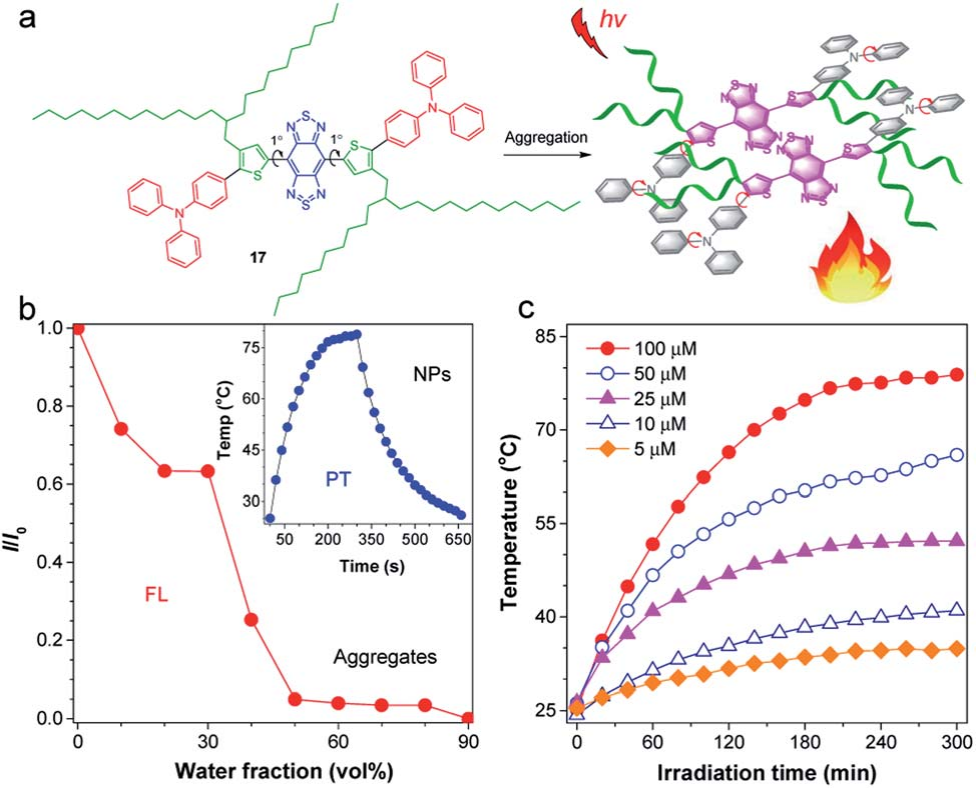
(a) Schematic illustration for activating intramolecular motion of 17 for boosted photothermal effect. (b) The plot of *I*/*I*_0_*versus* water fraction in H_2_O/THF mixtures. Inset: the photothermal property of 17 NPs. (c) Photothermal conversion behavior of 17 NPs at different concentrations (5–100 μM) under 808 nm laser irradiation. Data adapted with permission.^82^ Copyright 2019, American Chemical Society.

Increasing the photothermal conversion property of materials is crucial for their potential biological applications, as it reduces the incident light intensity, allowing for an efficient and safe therapy. Considering the dominated nonradiative decay in the TICT state, Tang and co-workers proposed a strategy for manipulating the TICT state to boost photothermal conversion property *via* activating molecular motion in aggregates.^82^ The molecular design guideline for an efficient photothermal agent is displayed in Fig. 7a. The central planar thiophene–BBTD–thiophene core (dihedral angle of 1°) was expected to quench the emission *via* strong intermolecular interactions when aggregated. The twisted TPA unit acted as molecular motors to assure intramolecular rotations and as a molecular donor to red-shift the absorption. The critical element of molecular design lies in the adoption of long-branched alkyl chains to provide the necessary room for molecular motions, which are the prerequisites for the formation of the TICT state.^83,84^ As expected, AIEgen 17 with 2-decylmyristyl unit displayed quenched fluorescence upon molecular aggregation (Fig. 7b). AIEgen 17-based NPs showed a high photothermal conversion efficiency (PCE) of 31.2%, better than the hexyl-decorated counterpart (22.6%), demonstrating a positive effect of long-branched alkyl chains for improving photothermal properties. Besides, the photothermal temperature was positively related to the concentration of NPs (Fig. 7c), which provided a personalized temperature selection for specific applications. Overall, activation of the intramolecular motions of D–A conjugated molecule in aggregates by twisted molecular motors and long-branched alkyl chains facilitated the formation of the dark TICT state to boost nonradiative pathway for heat generation.

**Fig. 8:**
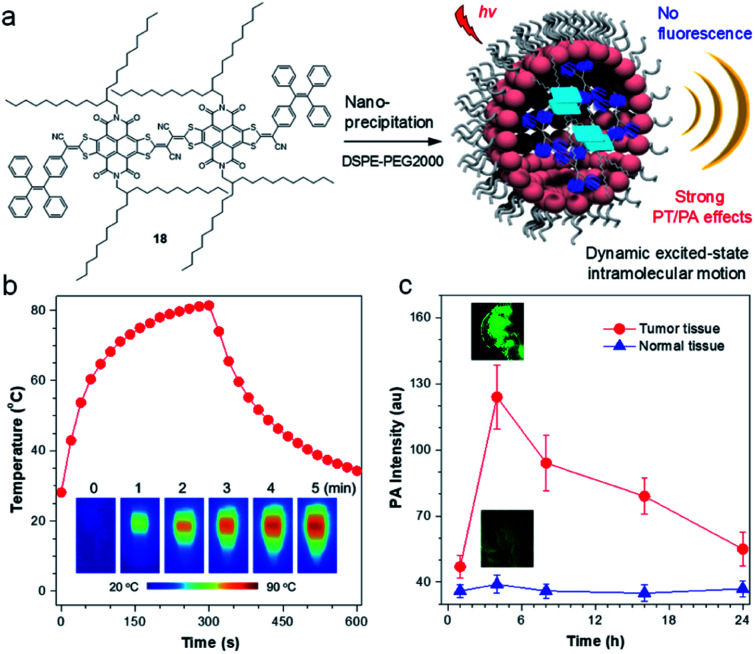
(a) Sketch map for the working mechanisms of iMIPT and the NPs of iMIPT molecule for PA imaging. (b) Temperature changes for the solutions of 18 NPs as a function of time upon irradiation with 808 nm laser (0.8 W cm^−2^) for 300 s. Inset: IR thermal images of 18 NPs upon exposure to 808 nm (0.8 W cm^−2^) laser irradiation for different times. (c) PA intensity of tumors and muscle tissues as a function of time before (0 h) and after intravenous injection of 18 NPs. Inset: PA images of tumors and muscles from 18 NPs-administrated mice after 18 NPs (300 μM based on 18) were intravenously injected into xenograft 4T1 tumor-bearing mice for 4 h. Data adapted with permission.^85^ Copyright, 2019, Nature Publishing Group.

To further illustrate the concept of intramolecular motion-induced photothermy (IMIPT), a molecule, 18, possessing a TPE molecular motor and long-branched alkyl chains in the π-conjugated planar backbone, was designed.^85^ Long-branched alkyl chains were aimed to achieve spatial isolation of molecules for enhanced molecular motions. The combination of π-expanded and strong electron-withdrawing acceptors with TPE (donor and twisted motor) contributed to not only a narrow energy gap beneficial for light-harvest ability, but also to trigger the TICT state giving a fluorescence quenching effect. Molecule 18 displayed excellent photothermal conversion property with temperatures rising to 81 °C (Fig. 8b). The PCE could even reach up to 54.9%, which is much higher than previous reports.^86^ The application of 18 NPs for *in vivo* PA imaging was then investigated. After intravenous injection of 18 NPs, a strong PA signal could be identified at the tumor site at 4 h post-injection, which is ∼2.7-fold higher than that of 0 h. As a control, the PA signal of the muscle tissues remained almost unchanged at the tested time points and was much lower than in the tumor.

Recently, based on the understanding of the strategies for activation of intramolecular motions within NPs, many excellent PTT/PA systems have been developed. Tang and co-workers reported an AIEgen full of molecular motors, allowing efficient intramolecular motions for PA imaging-guided phototherapy.^87^ Fan and co-workers boosted nonradiative decay channel through manipulating intermolecular charge transfer, attaining an exceptionally high PCE of 61%.^88^ Lee and co-workers reported a kind of π-conjugated oligomer F8-IC linked with A–D–A structure, whose intramolecular flexible rotations led to an enhanced non-radiative decay. The resulting F8-IC NPs displayed a high PCE of 82% for high-performance PA imaging-guided cancer therapy.^89^ Liu and co-workers integrated D–A structure and molecular motors into one molecule, TA1, giving a high PCE of 84.8%. The obtained TA1 NPs could significantly suppress primary breast tumor growth and metastasis.^90^ Peng and co-workers introduced a “barrier-free” motor to the *meso*-position of the BODIPY scaffold, allowing efficient dissipation of absorbed photons as heat. The obtained NPs showed a high PCE of 88.3% leading to complete removal of tumors in mice.^91^ Li and co-workers reported that the activation of intramolecular rotation around C=N double bond enabled a high PCE of ∼90%.^92^ Altogether, the activation of intramolecular motion within NPs provides a platform for developing efficient photothermal agents.

Despite high PCEs of above photothermal agents, they mainly rely on NIR-I laser. Owing to the deeper tissue penetration and higher maximum permissible exposure to the laser, photothermal agents in superior NIR-II region have received increasing attention.^93–95^ Nevertheless, pure organic materials with strong absorption in the NIR-II window have been far less reported, calling for high-performance materials with both long-wavelength absorption and high PCE.

## Conclusions

In this review, through regulation of hierarchical structures at a molecular level (flexibilization) and aggregate level (rigidification), respectively, organic NIR-II fluorophores with AIE characteristics for efficient fluorescence/photoacoustic imaging and PTT have been summarized. The molecular design strategy of AIEgens, “backbone distortion + motor twisting”, has been demonstrated to be indispensable for improving the QY of NIR-II dyes, due to the suppression of strong intermolecular interactions at aggregates. The flexibilization in molecular structure brings the fluorophores to a TICT state, giving a significantly red-shifted emission with strong NR. By intelligent control of AIE and TICT processes, molecular aggregates with high QY (AIE) and long-wavelength emission (TICT) can be achieved, which finds salient advantages for *in vivo* NIR-IIb imaging. On the other hand, systems can also display high fluorescence efficiency (AIE) with a tunable photothermal effect (TICT), which is suitable for fluorescence-guided PTT or fluorescence/photoacoustic dual-mode imaging. To further enhance the photothermal properties, a molecular guideline of “backbone planarization + motor/spacer” is proposed. The resultant fluorophores display an ACQ process owing to the intense intermolecular interactions from the planar conformation. However, with the presence of molecular motors and long-branched alkyl chain spacers, a significantly enhanced photothermal effect is detected owing to the activation of intramolecular motions. Overall, from molecule design to aggregate control, AIE research emphasizes the significance of studying materials properties at a higher hierarchical level.

While NIR-II AIEgens hold substantial promise for biological theranostics, several concerns need to be addressed for their further development. First, the overall brightness of NIR-II AIEgens can be further improved by increasing the molecular absorptivity while maintaining the high QY, as the backbone twisting in AIEgens may destroy the absorption. Second, pure organic fluorophores with peak emission exceeding the golden imaging region (>1500 nm) are seldom reported. Third, investigating ways to design NIR-II AIEgens with targeting ability or in response to external stimuli is of crucial importance. For example, Wu and Zeng reported a nitroreductase-responsive NIR-II AIEgen, realizing the detection of breast cancer metastasis.^96^ Last but not least, since many organic NIR-II NPs tend to accumulate in mononuclear phagocyte systems such as in livers and spleens, new NIR-II fluorophores should possess the overall dimension within the renal filtration limit (∼5 nm) for rapid urine excretion.

In summary, the development of single AIE NPs with balanced fluorescence and photothermal therapy functions through structure adjustment at different hierarchical levels is of great interest for the advancement of next-generation NIR-II fluorophores.

## Conflicts of interest

There are no conflicts to declare.

## Supplementary Material
